# CURL Extensions – Weaving a Unique Property Reference Number into Address Histories in the Scottish Community Health Index

**DOI:** 10.23889/ijpds.v9i5.2808

**Published:** 2024-09-18

**Authors:** David Clark

**Affiliations:** 1 Public Health Scotland

The Community Health Index (CHI) is the patient registration system for the National Health Service (NHS) in Scotland, covering the entire population. CHI has long been used as a population Indexing Spine to facilitate health-based data linkage for both operational and research purposes in Scotland.

Changes in patient’s residential address since 2000 have been captured in a CHI Address History File.

Addresses in CHI are recorded in unstructured free-text format, so property-based analyses and linkage is problematic. In August 2020, 90% of CHI addresses for currently registered patients, and people who had died that year, were seeded with a Unique Property Reference Number (UPRN). This product, called CHI-UPRN Residential Linkage (CURL), enabled NHS Scotland to harness important information on the patient population (5.8 million) and their place of residence, facilitating several data products related to COVID-19.

The current project is extending CURL to UPRN-seed the 31 million address histories of everyone registered with a family doctor in Scotland since January 2000.

The project, due for completion in Summer 2024, will look at alternative address-matching algorithms with the aim of achieving a 95% UPRN-match rate across the longitudinal CURL file.

Quality assurance exercises help us to understand the precision and sensitivity of matching across time and geography. Analysis of how the longitudinal file is constructed across the person and property-level indices permits a deeper understanding of the capabilities and limitations of the CURL product when used to deliver innovative analyses or modelling that link people and places over time.

